# Surgically and Conservatively Treated Obese Patients Differ in Psychological Factors, Regardless of Body Mass Index or Obesity-Related Co-Morbidities: A Comparison between Groups and an Analysis of Predictors

**DOI:** 10.1371/journal.pone.0117460

**Published:** 2015-02-13

**Authors:** Anne Ahnis, Andrea Figura, Tobias Hofmann, Andreas Stengel, Ulf Elbelt, Burghard F. Klapp

**Affiliations:** 1 Center for Internal Medicine and Dermatology, Division for General Internal and Psychosomatic Medicine, Charité—Universitätsmedizin Berlin, Berlin, Germany; 2 Center for Internal Medicine with Gastroenterology and Nephrology, Division for Endocrinology, Diabetes and Nutrition, Charité—Universitätsmedizin Berlin, Berlin, Germany; Vanderbilt University, UNITED STATES

## Abstract

**Objective:**

For the treatment of obesity, both conservative and surgical procedures are available. Psychological factors are likely to influence the choice of treatment; however, to date, systematic studies that investigate these factors are few in number. The aim of our study was to analyze whether patients who undergo a surgical treatment differ from those who require a conservative treatment in regard to psychological factors, regardless of their somatic conditions. Furthermore, predictors of treatment choice will be examined.

**Methods:**

A total of 244 patients (189 women), with a mean body mass index of 45.1 kg/m^2^, underwent a weight reduction treatment, with 126 patients undergoing bariatric surgery and 118 patients participating in a conservative, multimodal outpatient weight reduction program. Differences in the results of the psychological questionnaires between conservatively and surgically treated patients were evaluated through the use of t-tests, χ^2^-tests and an ANCOVA. For the analysis of the predictors, logistic regression models were calculated.

**Results:**

Surgically and conservatively treated obese patients differ in psychological, somatic, and socio-demographic factors. The psychological differences between the groups are independent of obesity-related co-morbidities, such as body mass index (BMI), type 2 diabetes mellitus, hypertension and coronary heart disease. The following psychological and somatic factors equally predict the choice of bariatric surgery: apathy, delegated active coping, a sense of coherence, complaints, type 2 diabetes mellitus, BMI, and age.

**Conclusion:**

Longitudinal studies are required to assess the predictive value of the psychological factors in regard to the postsurgical weight course to improve the pre-surgical screening and treatment selection process. The pre-surgical identification of psychological predictors should result in a more personalized medicine course and may ensure long term outcomes.

## Introduction

In addition to medical criteria (e.g., BMI and obesity-related co-morbidities), psychological factors may affect the choice of treatment method in obese patients and influence the course of the disease.

Few studies have analyzed the differences between patients who underwent bariatric surgery and those who requested surgery but were ineligible [1–3]. These studies did not identify differences between conservatively and surgically treated patients, with respect to age, gender or BMI [1–3]. The few studies that have analyzed psychological parameters in obese patients who underwent either conservative treatment or bariatric surgery [4–9] identified that the following variables are associated with patients who seek surgical treatment: higher BMI, younger age, higher levels of general psychopathology (CPRS Self-rating Scale for Affective Syndromes), higher levels of distress (obesity-related distress scale), lower perceived current health status (current health scale from the general health rating index), higher levels of health-related dysfunction in social life (social interaction category from the Sickness Impact Profile), higher psychosocial dysfunction due to obesity (obesity-related problems scale), obsessive symptoms (CPRS Self-rating Scale for Affective Syndromes), higher levels of aggression (irritability scale from the Karolinska Scales of Personality), less problem-oriented coping and more emotion-focused coping (obesity-related coping scale) [4–9].

However, these studies relied on a comparison of mean values, without controlling for BMI [except 9] or obesity-related co-morbidities (e.g., type 2 diabetes mellitus, hypertension, dyslipidemia), which may have caused the differences that were observed between conservatively and surgically treated patients. In addition, these studies were not able to comment on the predictive impact of psychological and somatic factors due to the chosen statistical method.

In contrast to previous studies, our study covers a wide range of psychological factors, including constructs that have not been examined, such as personal and social resources, physical discomfort and mood. Further, we examined psychological differences between surgically and conservatively treated obese women and men while controlling for the effect of the initial somatic conditions. Lastly, logistic regression analyses were employed to rank the relative importance of psychological, socio-demographic and somatic independent factors in regard to the choice of treatment.

## Methods

### Data selection

The present study investigated a sample of 244 obese patients who were treated between 12/2007 and 12/2011 at the Charité—Universitätsmedizin Berlin (Berlin, Germany). One hundred twenty-six obese patients were treated by surgical and endoscopic bariatric procedures, whereas 118 obese patients participated in a 1-year multimodal outpatient weight reduction program.

Obese patients whose primary focus was on surgical treatment initially visited the outpatient clinic of the Center of Obesity and Metabolic Surgery at the Charité—Universitätsmedizin Berlin, where they were examined, were advised and finally underwent the operation by the surgeon. Obese patients whose primary focus was on conservative treatment initially visited the outpatient clinic of the Division of Psychosomatic Medicine or the Metabolic Center of the Charité—Universitätsmedizin Berlin. The conservative treatment of a 1-year multimodal outpatient weight reduction program was offered. This program was advertised by a health insurance company and was mentioned on the homepage of the clinic. Patients were seen by a physician who specialized in psychosomatic medicine or a clinical psychologist and were included in the ambulant weight reduction program after they were examined to see whether they met the inclusion and exclusion criteria (A detailed description of the criteria for inclusion and exclusion, the evaluation procedure and its results is provided in the studies by Riedl et al. [10] and Ahnis et al. [11]).

According to the demands of the cost bearer in Germany (health insurance companies), patients who want to undergo bariatric surgery have to be evaluated by a psychiatrist, clinical psychologist or a physician who specializes in psychosomatic medicine to determine their mental condition. Therefore, the surgically treated patients also visited the Psychosomatic Division of the Charité—Universitätsmedizin Berlin after the consultation by the surgeon but before the bariatric operation.

Due to considerable social pressure, fear of exclusion or trends of denial on the patient's side, as well as preoccupation (and possibly prejudice) on the practitioner's side, we must assume that during the evaluation and treatment process, patients with an expectation of surgical treatment completed a conservative treatment program, and patients with an expectation of conservative treatment underwent bariatric surgery.

### Treatment procedures

Overall, 122 out of the 126 surgical patients underwent bariatric surgery, and 4 patients received a gastric balloon. The surgical, or endoscopic, procedures were performed by a single surgeon in accordance with the German guidelines for bariatric surgery [12]. Restrictive surgery was performed in 110 patients (10 received laparoscopic adjustable gastric banding, 100 received laparoscopic Sleeve Gastrectomy). Twelve patients underwent a laparoscopic Roux-en-Y Gastric Bypass, which is a restrictive-malabsorptive procedure.

The 1-year multimodal, conservative outpatient weight reduction program was divided into four areas of intervention and application: advice on diet and training, movement therapy and training, psychoeducation and behavioral therapy interventions, as well as Jacobson’s progressive muscle relaxation, which is detailed in previous reports [10, 11]. These interventions were conducted in group settings that were designed for 8 to 10 participants (and due to the initially high dropout rate that was expected, the program began with 12 to 14 participants) and were held twice a week for 2.5 hours during the first 6 months and once weekly for 2.5 hours during the second 6 months.

### Materials and statistical analyses

The data analysis was based on data that were collected during the initial visit at the Psychosomatic Division of the Charité—Universitätsmedizin Berlin prior to the surgical or conservative intervention. At the initial interview, the patient’s medical history was recorded, and clinical examinations were performed, including an evaluation of eating behavior and psychological tests (standardized questionnaires) that used personal digital assistants. Brief descriptions of the questionnaires are provided in **Table 1**. All patients provided written informed consent for the scientific use of their data. The evaluation procedure was approved by the ethics commission of the Charité—Universitätsmedizin Berlin; Application No EA 1/060/08.

**Table 1:** Overview and descriptions of the psychological tests that were employed.

Parameter	Questionnaire	Description
Socio-demographic parameters	SOZ—Questionnaire on social characteristics (German-language measure was used in hospital routines)	17 items assessing age, sex, occupational status, family status, etc.
Eating behavior	TFEQ—Three-Factor Eating Questionnaire [13] (German version: FEV—Fragebogen zum Essverhalten) [14]	66 Items assessing eating behavior, grouped into 3 scales: *Cognitive restraint of eating*, *Disinhibition*, *and Hunger*; Cronbach’s alpha = 0.54 to 0.76.
	EDI-2—Eating Disorder Inventory-2 (German version) [15]	64 items on 8 scales assessing specific psychopathologies of patients with anorexia and bulimia nervosa and other psychogenic eating disorders: *Drive for thinness*, *Bulimia*, *Body dissatisfaction*, *Ineffectiveness*, *Perfectionism*, *Interpersonal distrust*, *Interoceptive awareness*, *Maturity fears*; Cronbach’s alpha = 0.52 to 0.94.
Perceptions of stress	PSQ-20—Perceived Stress Questionnaire [16]	20 items assessing current subjective perceptions of stress, summarized on 4 scales that were entitled *Worries*, *Tension*, *Joy*, *and Demands*; Cronbach’s alpha = 0.82 to 0.94.
Complaints	GBB-24—Giessen Subjective Complaints List [17] (GBB-24, Giessener Beschwerdebogen)	24 items assessing various types of complaints, subdivided into 4 scales: *Exhaustion*, *Upper abdominal complaints*, *Limb complaints*, *Heart complaints* and the *total score of complaints (degree of complaints)*; Cronbach’s alpha = 0.79 to 0.94.
Psychic symptoms	ISR—ICD-10-Symptom-Rating [18, 19]	29 items assessing psychological symptoms, modeled on the syndromal approach of the ICD-10 and listed on 5 scales: *Anxiety syndrome*, *Obsessive-compulsive syndrome*, *Somatoform syndrome (Eating disorder syndrome and Depressive syndrome were not used in this study)*; Cronbach’s alpha = 0.74 to 0.92.
Depression	Depression scale of the PHQ—Patient Health Questionnaire (German version: PHQ—Gesundheitsfragebogen für Patienten) [20]	9 items assessing depression; Cronbach’s alpha = 0.88.
Mood	BMQ—Berlin Mood Questionnaire [21] (BSF, Berliner Stimmungsfragebogen)	30 Items on 6 scales assessing negative moods: *Tiredness*, *Apathy*, *Anxious-depressive mood*, *Anger;* and positive moods: *Involvement*, *Elated mood*; Cronbach’s alpha = .76 to .94.
Quality of life	SF-8—German version [22] of the SF-8 health survey [23]	8 items assessing health-related quality of life, the two total scores for *Mental health* and *Physical health* were used; Cronbach’s alpha = 0.42 to 0.60.
Resources	SWOP—Fragebogen zu Selbstwirksamkeit, Optimismus und Pessimismus (German questionnaire; assessment of beliefs in self-efficacy, optimism and pessimism) [24]	9 items assessing *Self-efficacy*, *Optimism* and *Pessimism* on 3 independent scales; Cronbach’s alpha = 0.67 to 0.83.
	SOC-9—German version of Antonovsky’s “sense of coherence” scale [25]	9 items assessing the *Sense of coherence*; Cronbach’s alpha = 0.89.
	PAS—Perceived Available Support, subscale of the Berlin social support scale [26]	8 items assessing *Perceived available emotional support* and *Perceived available instrumental support*; Cronbach’s alpha = 0.90 to 0.93.
Coping strategies	German version [27] of the Brief COPE [28]	28 items assessing coping behavior in past difficult or unpleasant situations, subdivided into 4 scales: *Seeking Support*, *Positive reframing*, *Avoidant coping* and *Delegated active coping (in original*: *Active coping)* ^*a*^; Cronbach’s alpha = 0.61 to 0.80.

^a^ We decided to rename the original scale of the Brief COPE “delegated active coping”, rather than “active coping” [27]. See discussion section for a detailed explanation.

For the descriptive statistical characterization, frequencies, means (*M*), standard deviations (*SD*), and ranges (*Min*, *Max*) were calculated for the psychological, somatic, and socio-demographic variables. A t-test for independent samples was employed to compare the means of the groups. Equality of variances, which was required for the t-test, was assessed by Levene’s test. In cases of differing variances, the test statistic *t* and error probability *p* were assessed based on the corrected degrees of freedom. The level of significance was set at *p* < .05. A chi-square four-field test was used for the nominally distributed variables. The magnitude of group differences was analyzed for the metrical data by Cohen´s *d* and for the nominal data by Cohen´s *w*. We used an analysis of covariance (ANCOVA) to control for the effects of the covariates, including BMI and obesity-related co-morbidities. Logistic regression analyses were employed to rank the relative importance of the psychological, socio-demographic and somatic independent variables in regard to the choice of treatment. To avoid over-fitting, the original number of independent variables was reduced. The variables that were identified as significant in the t-test and the chi-square test (after the correction of the alpha error by Bonferroni-Holm) were entered into a correlation matrix to test for multicollinearity, which would lead to estimation problems. Therefore, the variables with correlation values (Pearson’s *r* or Spearman’s *rho*) that were greater than 0.80 were eliminated. The calculated total test scores were excluded, or if the subscales had high internal correlations, the results for the subscales were removed, and the total score was included in the logistic regression model to avoid singularity (i.e., perfect collinearity). For all of the statistical analyses, the statistical software SPSS 20.0 for Windows (v 20.0; IBM Corp.; Armonk, NY) was used.

## Results

### Surgically and conservatively treated obese patients differ in socio-demographic and somatic factors

Surgically treated patients significantly differ from conservatively treated patients in certain socio-demographic variables: they are younger and more often male, have a lower educational level and are more often unemployed (**Table 2**).

**Table 2:** Socio-demographic differences between conservatively and surgically treated patients.

	Total number of patients^a^	Conservatively treated patients^b^	Surgically treated patients^c^	t- or *x*²-test		Effect size^d^
	M (SD)/N	M (SD)/N	M (SD)	t (df)/x² (df)	p	d / ω
Age in years	43.6 (11.5)	45.8 (11.7)	41.5 (11.0)	2.97 (242)	**.003** **	.38
Range	17–72	17–72	19–68			
Gender (f/m)	189/55	100/18	89/37	6.95 (1)	**.008** **	.17
Nationality^e^ (German/others)	229/7	115/1	114/6	3.51 (1)	.061	.12
Vocational education^e^ ^,^ ^f^ (High level vs. average/low level)	84/152	53/63	31/89	10.14 (1)	**.001** **	.21
Employed^e^ (yes/no)	152/89	89/26	63/63	19.34 (1)	**<.001** ***	.28
Partner relationship^e^ (yes/no)	149/91	66/48	83/43	1.62 (1)	.203	.08

** *p* < .01,

*** *p* < .001.

^a^ *N* = 237–244.

^b^ *N* = 114–118.

^c^ *N* = 121–126.

^d^ Cohen´s *d*: .20 = small effect, .50 moderate effect, .80 = large effect; Cohen´s *w*: .10 = small effect, .30 moderate effect, .50 = large effect

^e^ Employed: *N* = 3 not reported; partner relationship: *N* = 4 not reported; nationality and vocational education: *N* = 8 not reported.

^f^ A high level of vocational education includes university (degree of applied sciences) or technical college degree and being a student. An average or low level of vocational education degree includes full vocational status/completed vocational training, an apprenticeship, being a pupil, and no vocational education.

As indicated by **Table 3**, patients who underwent bariatric surgery weighed significantly more, had higher BMIs and consulted more physicians due to their current complaints than the conservatively treated patients did. Furthermore, patients who underwent bariatric surgery suffered significantly more often from type 2 diabetes mellitus, hypertension and coronary heart disease and less often from dyslipidemia.

**Table 3:** Somatic and disease-related differences between conservatively and surgically treated patients.

	Total number of patients^e^	Conservatively treated patients^f^	Surgically treated patients^g^	t- or *x*²-test		Effect size^h^
	M (SD)	M (SD)/N	M (SD)/N	t (df)/x² (df)	p	d / ω
Weight in kg	131.1(33.1)	113.1(26.5)	147.8(29.8)	-9.62(243)	**<.001** ***	-1.23
Range	72–251	72–208	98–251			
BMI in kg/m^2^	45.1(9.0)	39.5(6.8)	50.2(7.8)	-11.38(243)	**<.001** ***	**-1.46**
Range	30–78	30–62	35–78			
Doctor's certificate (forschool/work absence) last year (yes/no)	138/98	62/54	77/44	4.66(2)	.10	.18
1 month	84	42	42			
1–6 month	33	15	18			
> 6 month	21	5	16			
Duration of disease				5.40(4)	.25	.15
< ½ year	11	7	4			
½-1 year	10	6	4			
1–2 years	11	6	5			
2–5 years	28	18	10			
> 5 years	176	79	97			
Experience of psychotherapy (yes/no)	104/132	49/67	55/65	0.25(1)	.62	.05
< 1 month	9	3	6			
1–12 month	54	23	31			
> 12 month	41	23	18			
Number of consulted physicians due to the current complaints (1–2/≥3)	134/102	91/25	43/77	43.65(1)	**<.001** ***	**.43**
Type 2 diabetes mellitus (yes/no)	68/176	17/101	51/75	20.60(1)	**<.001** ***	**.29**
Hypertension (yes/no)	103/141	59/59	82/44	5.68(1)	**.017** *	**.15**
Dyslipidemia^a^ (yes/no)	88/156	56/62	32/94	12.86(1)	**<.001** ***	**.23**
Hyperuricemia (yes/no)	23/221	14/104	9/117	1.59(1)	.21	.08
Diseases of metabolic syndrome^b^ (0/1–4)	69/175	39/79	30/96	2.57(1)	.11	.10
Complete metabolic syndrome^c^ (yes/no)	9/225	5/113	4/122	0.19(1)	.66	.03
Coronary heart disease (yes/no)	22/222	4/114	18/108	8.82(1)	**.003** **	**.19**
Hyperphagic disorder^d^ (yes/no)	201/43	96/22	105/21	0.16(1)	.69	.03
Binge-eating disorder^d^ (yes/no)	28/216	15/103	13/113	0.34(1)	.56	.04

* *p* < .05,

** *p* < .01,

*** *p* < .001.

^a^ hypercholesterolemia, hypertriglyceridemia, combined hyperlipidemia, disorder of HDL, metabolism (isolated low HDL)

^b^ type 2 diabetes mellitus, hypertension, dyslipidemia, hyperuricemia

^c^ without obesity

^d^ clinical interview by specialist in psychosomatic medicine at initial presentation

^e^ *N* = 237–244

^f^ *N* = 116–118

^g^ *N* = 121–126

^h^ Cohen´s *d*: .20 = small effect, .50 moderate effect, .80 = large effect; Cohen´s *w*: .10 = small effect, .30 moderate effect, .50 = large effect

The following variables/items were not reported by 8 patients: doctor’s certificate, duration of disease, experience of psychotherapy, and the number of consulted physicians due to the current complaints.

### Surgically and conservatively treated obese patients differ in psychological factors, independent of their somatic conditions

Compared to patients who chose conservative therapy, the patients who chose bariatric surgery had less favorable scores on almost all of the psychological variables (**Table 4**). Regarding their eating behavior, they reported more “perceived feelings of hunger” (TFEQ), more “drive for thinness” (EDI-2) and more “ineffectiveness” (assesses feelings of inadequacy, insecurity, worthlessness and lack of control over their lives) (EDI-2). Regarding stress and complaints, they reported more “perceived stress”, less “joy” (PSQ) and higher scores for “complaints” overall and on all of the dimensions of singular complaints (GBB-24). Regarding mood, psychological symptoms and quality of life, they reported more negative and less positive “mood” (BMQ), more psychopathology, with a higher total symptoms score (ISR), and higher subscores for “anxiety syndrome” or “somatoform syndrome”. On the PHQ, they reported more “depression”, as well as less “mental health” and “physical health” (SF-8). Regarding their resources and coping strategies, they had less “sense of coherence” (SOC), more “pessimism” (SWOP) and more “avoidant coping” and “delegated active coping” (Brief COPE).

**Table 4:** Group differences in psychological variables between conservatively and surgically treated patients.

	Norm samples^c^	Conservatively treated patients^a^	Surgically treated patients^b^	t-test / Effect size			ANCOVA			
	M (SD)	M (SD)	M (SD)	t (df)	p (two-tailed)	d	p (BMI-adjusted)	p (diabetes-adjusted)	p (hypertension-adjusted)	p (CHD^d^-adjusted)
TFEQ										
Restraint	8.2 (5.0)	9.4 (4.6)	9.5 (5.5)	-0.16 (192)	.87	-.02				
Disinhibition	7.1 (3.9)	8.8 (3.6)	9.3 (4.0)	-0.85 (192)	.40	-.13				
**Hunger**	**5.7 (3.4)**	**6.5 (3.8)**	**8.1 (4.3)**	**-2.68 (192)**	**.008** **	**-.39**	.057	**.012** *	**.021** *	**.010** *
**EDI-2**										
**Total score**	**n. a.**	**186.9 (40.0)**	**208.1 (48.7)**	**-3.37 (197)**	**.001** **	**-.48**	**.14**	**.004** **	**.004** **	**.003** **
**Drive for thinness**	**17.3 (6.8)**	**24.7 (7.2)**	**29.2 (7.5)**	**-4.29 (197)**	**<.001** ***	**-.61**	**.001** **	**<.001** ***	**<.001** ***	**<.001** ***
Bulimia	10.6 (3.4)	15.2 (6.9)	17.0 (7.4)	-1.73 (197)	.086	-.25				
Body dissatisfaction	30.2 (10.3)	44.9 (9.0)	47.1 (10.2)	-1.59 (197)	.11	-.23				
**Ineffectiveness**	**23.5 (5.7)**	**23.6 (8.7)**	**27.9 (9.9)**	**-3.28 (197)**	**.001** **	**-.46**	.28	**.005** **	**.006** **	**.003** **
Perfectionism	16.5 (5.7)	16.8 (6.1)	17.9 (6.4)	-1.19 (197)	.24	-.18				
Interpersonal distrust	18.4 (4.4)	19.1 (6.3)	21.2 (7.3)	-2.15 (197)	.033*	-.31				
Interoceptive awareness	22.0 (5.7)	21.9 (7.3)	25.2 (9.1)	-2.70 (154.90)	.008**	-.40				
Maturity fears	20.8 (4.7)	20.7 (5.1)	22.8 (6.6)	-2.49 (197)	.014*	-.36				
**PSQ**										
**Total score**	**0.3 (0.2)**	**0.4 (0.2)**	**0.5 (0.2)**	**-3.53 (242)**	**<.001** ***	**-.50**	**.001** **	**.002** **	**.002** **	**.002** **
General demands	0.4 (0.1)	0.4 (0.2)	0.4 (0.2)	0.04 (242)	.97	.00				
Tension	0.4 (0.1)	0.4 (0.3)	0.5 (0.2)	-2.66 (242)	.008**	-.39	.007**	.031*	.031*	.019*
Worries	0.3 (0.2)	0.4 (0.2)	0.5 (0.3)	-4.20 (242)	<.001***	-.39	<.001***	<.001***	<.001***	<.001***
Joy	0.6 (0.2)	0.6 (0.3)	0.4 (0.2)	4.97 (233.51)	<.001***	.78	.001**	<.001***	<.001***	<.001***
**GBB-24**										
**Total score**	**14.0 (12.7)**	**23.4 (14.8)**	**37.2 (17.1)**	**-6.75 (242)**	**<.001** ***	**-.86**	**<.001** ***	**<.001** ***	**<.001** ***	**<.001** ***
**Exhaustion**	**3.9 (4.0)**	**7.1 (5.5)**	**11.5 (5.8)**	**-6.11 (241)**	**<.001** ***	**-.78**	**<.001** ***	**<.001** ***	**<.001** ***	**<.001** ***
**Limb complaints**	**5.4 (4.6)**	**9.7 (5.3)**	**13.9 (5.2)**	**-6.13 (242)**	**<.001** ***	**-.80**	**<.001** ***	**<.001** ***	**<.001** ***	**<.001** ***
**Upper abdominal complaints**	**2.6 (3.2)**	**3.4 (3.4)**	**5.6 (4.4)**	**-4.23 (232.48)**	**<.001** ***	**-.56**	**<.001** ***	**<.001** ***	**<.001** ***	**<.001** ***
**Heart complaints**	**2.2 (3.2)**	**3.1 (3.5)**	**6.3 (5.1)**	**-5.72 (221.69)**	**<.001** ***	**-.73**	**<.001** ***	**<.001** ***	**<.001** ***	**<.001** ***
**ISR**										
**Total score**	**0.4 (0.5)**	**0.7 (0.5)**	**1.1 (0.6)**	**-4.96 (238.90)**	**<.001** ***	**-.72**	**<.001** ***	**<.001** ***	**<.001** ***	**<.001** ***
**Anxiety syndrome**	**0.5 (0.7)**	**0.7 (0.8)**	**1.1 (1.0)**	**-3.16 (230.10)**	**.002** **	**-.44**	.053	**.007** **	**.002** **	**.005** **
Obsessive-compulsive syndrome	0.3 (0.6)	0.5 (0.8)	0.6 (0.8)	-0.81 (241)	.42	-.12				
**Somatoform syndrome**	**0.4 (0.6)**	**0.4 (0.6)**	**0.7 (0.9)**	**-3.36 (222.94)**	**.001** **	**-.39**	**.014** *	**.002** **	**.001** **	**.004** **
**PHQ-9 (Depression)**	**3.6 (4.1)**	**6.3 (5.3)**	**9.5 (5.6)**	**-4.67 (241)**	**<.001** ***	**-.59**	**.001** **	**<.001** ***	**<.001** ***	**<.001** ***
**BMQ**										
**Elated mood**	**n. a.**	**1.7 (1.1)**	**1.3 (1.0)**	**3.07 (241)**	**.002** **	**.38**	**.009** **	**.002** **	**.010** *	**.001** **
**Involvement**	**n. a.**	**2.3 (0.8)**	**2.1 (0.7)**	**2.45 (241)**	**.014** *	**.27**				
**Anger**	**n. a.**	**0.5 (0.6)**	**0.8 (0.8)**	**-3.69 (226.79)**	**<.001** ***	**-.42**	**.008** **	**.001** **	**.001** **	**.001** **
**Anxious-depressive mood**	**n. a.**	**1.0 (0.9)**	**1.4 (1.0)**	**-3.63 (238.84)**	**<.001** ***	**-.42**	**.003** **	**.002** **	**.002** **	<.001***
**Tiredness**	**n. a.**	**1.2 (1.0)**	**1.8 (1.1)**	**-4.34 (242)**	**<.001** ***	**-.57**	**<.001** ***	**<.001** ***	**<.001** ***	**<.001** ***
**Apathy**	**n. a.**	**0.3 (0.6)**	**0.8 (0.7)**	**-5.12 (230.36)**	**<.001** ***	**-.77**	**.001** **	**<.001** ***	**<.001** ***	**<.001** ***
**SF-8**										
**Mental health**	**49.2 (9.5)**	**48.5 (11.9)**	**44.6 (12.1)**	**2.53 (237)**	**.012** *	**.32**	**.044** *	**.032** *	**.034** *	**.001** **
**Physical health**	**49.2 (9.1)**	**41.4 (10.9)**	**31.9 (9.4)**	**7.24 (237)**	**<.001** ***	**.93**	**<.001** ***	**<.001** ***	**<.001** ***	**<.001** ***
**PAS**										
Perceived available emotional support	n. a.	13.8 (2.6)	13.7 (2.7)	0.24 (237)	.86	.04				
Perceived available instrumental support	n. a.	13.5 (2.8)	13.5 (3.0)	-0.03 (237)	.98	.00				
**SOC (Sense of coherence)**	**5.3 (n. a.)**	**5.1 (1.1)**	**4.8 (1.2)**	**2.10 (239)**	**.038** *	**.26**	.51	.11	.12	**.033** *
**SWOP**										
Self-efficacy	2.8 (0.7)	2.8 (0.6)	2.7 (0.6)	1.20 (239)	.23	.17				
Optimism	2.8 (0.9)	2.9 (0.8)	2.7 (0.8)	2.20 (239)	.030*	.25				
**Pessimism**	**2.2 (0.8)**	**2.0 (0.7)**	**2.3 (0.8)**	**-2.99 (239)**	**.003** **	**-.40**	**.025** *	**.012** *	**.012** *	**.010** *
**Brief COPE**										
**Avoidant coping**	**1.4 (0.5)**	**2.0 (0.5)**	**2.2 (0.5)**	**-3.04 (239)**	**.003** **	**-.40**	**.010** *	**.009** **	**.011** *	**.01** *
Seeking support	1.9 (0.6)	2.1 (0.5)	2.2 (0.5)	-2.09 (239)	.038*	-.20				
Positive reframing	2.4 (0.7)	2.0 (0.5)	1.9 (0.5)	0.88 (239)	.38	.20				
**Delegated active coping**	**2.1 (0.8)**	**2.8 (0.7)**	**3.2 (0.6)**	**-4.61 (239)**	**<.001** ***	**-.61**	**<.001** ***	**<.001** ***	**<.001** ***	**<.001** ***

* *p* < .05

** *p* < .01

*** *p* < .001. Correction of the alpha error for each psychometric test, as described by Bonferroni-Holm (value marked in boldface = significant after correction).

^a^ *N* = 111–118

^b^ *N* = 83–126

^c^ Norm samples: TFEQ: *N* = 1097 women with and without weight problems, age: *M* = 30.1, BMI: *M* = 22.8 [14]; EDI-2: *N* = 186 general population (women), age: *M* = 28, BMI: *M* = 22 [15]; PSQ: *N* = 246 medical students, age: *M* = 24.6 [16]; GBB-24: *N* = 2182 general population, age = *M* = 39.4 [29]; ISR: *N* = 2512 general population, age: *M* = 49 [30]; PHQ-9: *N* = 2063 general population, age: *M* = 48.8 [31]; SF-8: *N* = 7472 general population [23]; SOC: *N* = 700 general population, age: 41–60 [25]; SWOP: *N* = 726, age: *M* = 45.3 [24]; Brief COPE: *N* = 94 of 110 cataract patients, age: *M* = 71.6 [27].

^d^ Coronary heart disease

Abbreviations: n. a., not available; TFEQ, Three-Factor Eating Questionnaire [13] (German version: FEV [14]); EDI-2, Eating Disorder Inventory-2 (German version [15]); PSQ, Perceived Stress Questionnaire [16]; GBB-24—Giessen Subjective Complaints List [17] (GBB-24, Giessener Beschwerdebogen); ISR, ICD-10-Symptom-Rating [18, 19]; Brief PHQ, Brief Patient Health Questionnaire (depression scale, PHQ-9) [20]; BMQ—Berlin Mood Questionnaire [21] (BSF, Berliner Stimmungsfragebogen); SF-8, German version of the Health Survey [22]; PAS, Perceived Available Support, subscale of the Berlin Social Support Scale [26]; SOC, Sense of Coherence Scale [25]; SWOP, Fragebogen zu Selbstwirksamkeit, Optimismus und Pessimismus [24] (Assessment of Beliefs in Self-Efficacy and Optimism); Brief COPE [28], German version of the Brief COPE [27].

After controlling for the confounding factors of BMI and type 2 diabetes mellitus, hypertension and coronary heart disease, the psychological differences persisted between the groups **(Table 4)**, which indicates that the differences are independent of these somatic conditions.

### Somatic, as well as psychological, factors predict the choice of treatment

The predictive value of the investigated somatic, psychological and socio-demographic variables in regard to the choice of treatment method (bariatric surgery, reference category: conservative treatment) was determined by a logistic regression analysis, with the following independent variables being entered into the regression model: BMI, type 2 diabetes mellitus (reference category: no type 2 diabetes mellitus), hypertension (reference category: no hypertension), dyslipidemia (reference category: no dyslipidemia), coronary heart disease (reference category: no coronary heart disease), anxiety syndrome (ISR), degree of complaints (GBB-24), expression of anger and apathy (BMQ), mental health and physical health (SF-8), sense of coherence (SOC), pessimism (SWOP), depression (PHQ), avoidant and delegated active coping (Brief COPE), age, gender (reference category: female), work (reference category: employment), vocational education (reference category: high degree of vocational education) and the number of physicians who were consulted due to their current complaints (reference category: 1–2 consulted physicians).

The main outcome of the binary logistic regression is that certain somatic and psychological factors equally predict the choice of bariatric surgery.

As indicated in **Table 5**, a unit change in BMI increases the odds of the event of bariatric surgery = 1 approximately 1.5 times. The odds of being surgically treated, compared to being conservatively treated, are increased by a factor of 54.34 when the patient suffers from type 2 diabetes mellitus, after controlling for other variables in the model. A one unit change on the “apathy” scale (BMQ) increases the odds of the event bariatric surgery = 1 approximately 47.2 times, after controlling for other variables in the model. A one unit change on the “degree of complaints” scale (GBB-24) increases the odds of being surgically treated by a factor of 1.15. The odds of being surgically treated, compared to being conservatively treated, increase by a factor of 8.35 for one unit change on the “sense of coherence” scale (SOC), after controlling for other variables in the model. A one unit change on the “delegated active coping” scale (Brief COPE) increases the odds of the event of bariatric surgery = 1 approximately 28.52 times. Finally, the odds of being surgically treated, compared to being conservatively treated, decreased by a factor of 0.9 for each year the age increased, after controlling for other variables in the model.

**Table 5:** Predictors for bariatric surgery^a^, which are determined by a logistic regression analysis.

	Regression coefficient	Standard-error	Significance	Odds ratio	95% Confidence Interval for *Exp(B)*	
Explanatory variable	B	SE	p	Exp(B)^b^	Lower Bound	Upper Bound
Type 2 diabetes mellitus^c^	4.00	1.12	<.001	54.34	6.11	483.02
Apathy (BMQ)	3.85	1.07	<.001	47.17	5.85	380.59
Delegated active coping (Brief COPE)	3.35	0.85	<.001	28.52	5.39	150.83
Sense of coherence (SOC)	2.12	0.69	.002	8.35	2.15	32.51
BMI	0.41	0.09	<.001	1.51	1.27	1.79
Degree of complaints (GBB-24)	0.14	0.04	.001	1.15	1.06	1.25
Age	-0.16	0.05	.001	0.85	.77	.94
Dyslipidemia^d^	-1.83	0.80	.022	0.16	.03	.77

The dataset was reduced from *N* = 244 cases to *N* = 226 cases due to missing data. 5 cases (of *N* = 226) were identified as outliers by having a Pearson’s residual (*z residual*) > 3 and were excluded from the regression analysis. For the calculation of the model (cases: *N* = 221), all of the selected variables were entered simultaneously. Only the significant variable effects are shown. Omnibus test of model coefficients: *x* ^*2*^ = 223.86, *df* = 21, *P* < .001. Nagelkerke`s *R* ^*2*^ = 0.85. Analysis of the classification results: groups were not equally distributed; 90.5% of cases had been correctly predicted/classified (surgical patients: 91.4, conservatively treated patients: 89.5%).

^a^ reference category: conservative treatment

^b^ The *Exp(B)* (effect coefficients) show the delogarithmized logit coefficients as odds ratios. *Exp(B)* = 1.0: the independent variable has no effect. *Exp(B)* < 1: the independent variable decreases the logit and, therefore, decreases the odds (of bariatric surgery) (marked in italics). *Exp(B)* > 1: the independent variable increases the logit and increases the odds (of bariatric surgery) (marked in bold type).

^c^ reference category: no type 2 diabetes mellitus

^d^ reference category: no dyslipidemia

### Discussion

In the present study, we found a great number of differences between surgically and conservatively treated obese patients regarding the psychological, somatic and socio-demographic factors. We demonstrated that psychological differences between the two groups persisted, even after controlling for BMI and obesity-related co-morbidities. This result relativized previous assumptions that psychological differences between conservatively and surgically treated patients can only be attributed to somatic differences. The higher prevalence of type 2 diabetes, arterial hypertension and coronary heart disease, which we found in surgically treated patients, compared to conservatively treated patients, could be explained by the more frequent occurrence of those diseases in more severely obese subjects (also see [32, 33]). Consistent with previous research [4, 5, 8] and as determined by the German guidelines for bariatric surgery (BMI of ≥ 40 kg/m² or BMI between 35 and 40 kg/m² for patients with a serious obesity-associated co-morbidity, [12]), our surgically treated patients exhibited a higher (initial) weight and higher BMI.

However, dyslipidemia was observed less often in those who sought bariatric surgery. We assume that this diagnosis is observed more frequently in our conservatively treated patients because a large proportion of these patients (*N* = 46 out of 118) was referred from the Metabolic Center at the Charité—Universitätsmedizin Berlin, where patients are initially treated for dyslipidemia. Accordingly, this result may be due to the data selection process.

Further we showed that psychological variables (i.e., delegated active coping, sense of coherence, apathy, complaints) and the socio-demographic variable of age, as well as somatic variables, predict the choice of bariatric surgery.

The surgically treated patients had significantly worse physical conditions before the intervention. They may expect that surgical treatment will result in a rapid weight loss and pain relief. Another possible interpretation is that patients who request surgical treatment, compared to conservatively treated patients, may tend to more strongly act out conflicts and the related negative emotions on a somatic level. Regarding the clinical implications in this case, it remains doubtful that bariatric surgery alone is a sufficient intervention strategy.

Ryden et al. [6] reported that patients who underwent bariatric surgery, compared to conservatively treated obese patients, exhibited more emotional and less problem-oriented coping strategies. Interestingly, and in contrast to these findings, our surgically treated patients exhibited higher scores on the (delegated) active coping scale, which is interpreted by Carver [28] (in the original version of the Brief COPE) as a problem-oriented approach to difficult situations. The items on the (delegated) active coping scale include the following: "I have been concentrating on changing my situation"; "I actively acted to improve the situation”; “I have thought a lot about what would be the right thing to do”; and “I have tried to make a plan”. Nevertheless, in our study, the high value on this scale should be interpreted differently than Carver et al. [28] and Knoll et al. [27] originally intended. In addition to participating in numerous necessary preliminary examinations (i.e., surgical, psychosomatic, endocrinological), which are reflected in the higher number of physicians (*N* ≥ 3) who are consulted before intervention (also see [34]), patients who request surgery are mandated to participate in a patient information event in our center (for approval and cost coverage by the patients’ health insurance company, a recommendation for bariatric surgery by the multidisciplinary team is necessary). During the patient information event, patients are informed about causal factors and treatment options for obesity and have the opportunity to clarify additional issues or questions. Additionally, patients are invited to participate in support group meetings. Patients must submit the application for reimbursement for bariatric surgery to the insurance company, and often, they have to respond to a rejection by the health insurance company and explain the situation. These activities may be interpreted by the patient as actively coping with their disease. However, strictly interpreted, these activities do not reflect behavioral changes that occur to reduce body weight (e.g., increasing physical activity, which we demonstrate in [35]). These activities indicate the patients' submission to the recognized rules of the health insurance company and medical system, with which the patient attempts to cooperate to obtain what he or she wants (i.e., a delegation of activity to the medical system). Therefore, we decided to rename the original scale of the Brief COPE “delegated active coping” rather than “active coping” [27]. The interpretation of delegating responsibility is supported by the surgical patients' higher scores on the "avoidant coping" scale, compared to the conservatively treated patients' scores (e.g., "I have told myself that everything/this is not true"; "I have been open about how badly I feel"; "I have been blaming myself for things that have happened to me”). Additionally, for our surgically scheduled patients, the dimensions of "avoidant coping" and "delegated active coping" have a slight positive correlation before intervention (*N* = 126, *r* = 0.20, *p* < .05).

The mood variable of “apathy” (BMQ) (e.g., “I feel uninvolved/uninterested/indifferent/bored.”) also proved to be a predictor of bariatric surgery. In certain ways, this passivity is reflected in the choice of the surgical approach, which is a predominantly passive treatment for the patient. In regard to the clinical implication, the question remains whether it is good that apathetic patients are more often referred to bariatric surgery. On the one hand, it may be the only solution for them; on the other hand, the post-surgical compliance may be low.

In our regression analysis, “sense of coherence” proved to be a predictor of preferring bariatric surgery, whereas the t-test indicated that the variable was expectedly higher in conservatively treated patients. However, this effect disappeared after controlling for BMI, type 2 diabetes mellitus and hypertension. In the regression analysis, the effect inverted when all of the other variables were held constant. To understand this result, in additional regression models, we tried to identify the variables that led to a reversal of the effect. We found that the effect inverted when the variables of “degree of complaints” (GBB-24) and “apathy” (BMQ), as well as “degree of complaints” (GBB-24) and “depression” (PHQ), were held constant. This may indicate that an overall more coherent and less fatalistic picture is created for certain patients when they believe that surgery provides a solution to their weight problem. However, this only occurs in those patients who rarely complain and who do not feel apathetic or depressed.

Surgically and conservatively treated patients did not differ in the prevalence of hyperphagic eating disorder, binge eating disorder, or other specific eating behaviors, which was shown by means of the EDI-2 and TFEQ questionnaires (also see [9]). This lack of a difference may be due to a ceiling effect: when a specific BMI is reached, an eating disorder or a pathological eating behavior cannot become more pronounced. We only observed differences in the eating disorder questionnaires on the subscales of "hunger" (TFEQ) (also see [9]), "drive for thinness" (an excessive concern with dieting, preoccupation with weight, and fear of weight gain) and "ineffectiveness" (assesses feelings of inadequacy, insecurity, worthlessness and lack of control over their lives) (EDI-2). These scales are not representative of eating behavior; rather, they are representative of subjective feelings of hunger/satiety, one's attitude towards the body and weight, and one's experience of inadequacy or a reduction in self-worth.

Certain methodological and statistical limitations of the outcome should be kept in mind. First, the current study was a retrospective analysis of data that were collected before the intervention for the psychological, socio-demographic and somatic variables to potentially predict the preference of bariatric surgery. Second, generalization to other Western countries may be limited due to the patients' adherence to the German guidelines for bariatric surgery. Third, the nature of self-reports should be interpreted with caution because a social desirability bias may affect the results. This possibility is currently being discussed among bariatric-surgical expert groups, with regard to the inclusion and exclusion criteria for surgical treatments for obesity. Thus, they may find that the prevalence figures regarding the comorbidity of certain psychological aspects in severely obese patients were underestimated (Gruß, 2010, congress contribution in [36]). This may be related to the fact that the psychological diagnostic evaluation is one major part of the pre-surgical assessment and that patients who are aware of its significance did not report mental symptoms to obtain coverage for the bariatric intervention by their health insurance. Lastly, the low variance in certain continuous predictor variables and sparse cell data for a few of the categorical predictors may cause the odds ratio to be overestimated in the logistic regression analysis. Large odds ratios could also be due to unobserved confounders related to the non-randomized study design.

In summary, the current study identified that psychological factors are independent of somatic conditions in obese patients who seek a surgical, rather than a conservative, weight reduction treatment. The predictive value of a few of the psycho-social factors in regard to the treatment choice was proven. Additional studies, particularly longitudinal studies, are required to assess the predictive value of psychological factors on the postsurgical weight course and improve the pre-surgical screening and treatment selection process. Our research will focus on the extent to which already identified predictors influence post-operative weight loss when we investigate the follow-up data regarding the patients' situation after the bariatric surgery. The identification of predictors that can be therapeutically addressed before surgery to secure sufficient and sustained weight loss after the bariatric surgery is essential when determining treatment pathways for patients and may result in a more personalized medicine course.
